# Like a Fish out of Water: Temporary Habitat Switching Detected in Aquatic Tadpoles Resting Above the Water's Surface

**DOI:** 10.1002/ece3.71057

**Published:** 2025-03-30

**Authors:** John Gould, Claire Larkin, Colin McHenry

**Affiliations:** ^1^ School of Environmental and Life Sciences University of Newcastle Callaghan New South Wales Australia

**Keywords:** air‐breathing, anuran, *Azolla*, duckweed, hypoxic water, predator escape

## Abstract

Amphibian tadpoles are typically aquatic and constrained to freshwater throughout development. These circumstances change at metamorphosis, whereupon individuals may complete a partial or full habitat shift towards terrestriality and gain the capacity to transition between habitat types. In this study, we present evidence of striped marsh frog tadpoles, *Limnodynastes peronii* , resting out of the water on floating vegetation mats in freshwater ponds, despite being fully aquatic and at a developmental stage far proceeding metamorphosis. We propose that this behaviour may represent a form of adaptive beaching to obtain survival benefits that are derived from being out of the water column. In particular, this may be a behavioural tactic of habitat switching to avoid aquatic predators, such as the introduced mosquitofish, *Gambusia holbrooki* , or to access oxygen from the air when the water becomes hypoxic. The capacity for aquatic tadpoles to exploit temporary refuges outside the water challenges traditional assumptions about their strict dependence on being continuously submerged below the surface for survival. While our findings are preliminary and based on a small sample size, they suggest the aquatic tadpoles of our focal species and perhaps others are not as restricted to their natal waterbodies as previously thought and that they have the capacity to move into habitats above the water's surface for resource gain.

## Graphical abstract

Habitat switching is a behavioural or developmental process by which animals move from one habitat type to another for fitness gain. We provide observations of tadpoles of the striped marsh frog, *Limnodynastes peronii*, resting out of the water column on floating aquatic vegetation, despite being fully aquatic and at a developmental stage far proceeding metamorphosis. Given that the observed tadpoles were able to descend back into the water column when disturbed, we suggest that aquatic tadpoles of some species may participate in habitat switching behaviour, being able to move out of the water temporally for a benefit that may include predator avoidance or respiration.

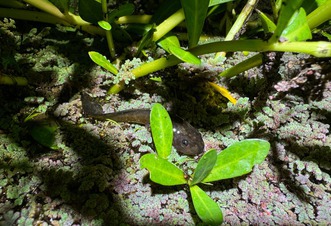

## 1 Introduction

Temporal habitat shifts are widely displayed by animals within and between life stages as it can provide fitness benefits in terms of survival and reproduction (Nolet et al. 2002). These shifts are referred to as habitat switching and can be (1) behavioural and occur within a particular life stage, such as seasonal migrations (Dawbin 1966), or (2) developmental and occur between life stages, such as the abrupt changes in habitat use with metamorphosis (Wells 2007). Habitat switching may also comprise relatively smaller, temporary, and periodic transitions, such as movement back and forth between habitats driven by predator avoidance or foraging (Ansari et al. 2014). These movements allow individuals to exploit habitats while they are still profitable in terms of resource availability (e.g., nutrients) until depleted to a critical level (Rowcliffe et al. 2001), or to accommodate changes in resource needs when resources are spatially isolated (Dawbin 1966), or to temporarily escape sub‐optimal conditions before returning (Vila et al. 2008). Individuals must be able to respond appropriately to environmental cues that trigger habitat switching in order for this process to improve the odds of reward and for such movements to outweigh the risks of remaining put.

Amphibians have a complex life cycle that typically involves the external deposition of eggs into freshwater, which then hatch into tadpoles that are free‐swimming, aquatic, and feed on external food sources (i.e., exotrophic) (Duellman and Trueb 1986). Upon hatching, aquatic tadpoles are effectively constrained to the water column until metamorphosis is achieved, whereafter they acquire the ability to escape into the surrounding terrestrial environment (Wells 2007). Aquatic tadpoles must be almost continuously immersed in water to (1) prevent desiccation, given their permeable skin that can easily lose moisture (Venturelli et al. 2021), and (2) breathe by extracting oxygen from the water using their gills and skin (Phillips et al. 2020; Wells 2007). Their need for free‐standing water and resulting vulnerability to its absence is highlighted by cases of mass tadpole mortality that can occur if clutches are laid in ephemeral waterbodies that dry too rapidly before tadpole development can be successfully completed (Gould et al. 2022); although the tadpoles of some species may show relatively higher yet still only temporary resistance to desiccation when out of water (Venturelli et al. 2021). This is in contrast to those species of amphibian with direct development that lack a free‐swimming tadpole stage (Callery et al. 2001), ‘endotrophic’ tadpoles that hatch at an advanced stage and complete development without feeding (Thibaudeau and Altig 1999), and some dendrobatids whose parents temporarily carry their tadpoles on their backs (Downie et al. 2005); in these cases, the reliance on free‐standing water and waterbodies is partially or fully reduced.

Another critical threat to amphibian tadpoles while confined to the water column is the presence of aquatic predators such as macroinvertebrates and fish (Beranek et al. 2021; Gould et al. 2019; Pyke and White 2000). Despite being mobile and thus able to swim away from potential threats, there is often no means by which tadpoles can move out of their natal aquatic systems to avoid them completely. This is because tadpoles generally do not have the capacity to move across land like other aquatic animals that are also dependent on free‐standing water (e.g., eels; Gillis 1998), with exceptions being during periods of flooding and the formation of water channels that connect neighbouring aquatic systems (Rivero 1961). For amphibian parents, choosing the right oviposition site is thus critical for increasing the chance of offspring survival. Yet, it may not be possible for parents to detect threats if they arise later during development and cannot be accurately predicted at the moment of egg deposition (Gould et al. 2022). In these scenarios, tadpoles are entirely reliant on tactics to protect themselves from threats while remaining in the water. Strategies to reduce desiccation risk include rapid and/or plastic developmental rates (Delgadillo Méndez et al. 2024), while predation risk can be reduced by seeking refuge in aquatic microhabitat, adjusting activity levels and periods, and cryptic colouration (Bridges 2002; Calef 1973; Espanha et al. 2016).

While aquatic tadpoles with a generalised life history are primarily confined to the water column, it is apparent that they can be removed from water for short periods without succumbing to desiccation‐related injuries or lack of oxygen (e.g., Gould et al. 2023). Resistance to hydric stress has also been shown in the field for tadpoles of several species, including aquatic tadpoles that can survive hours or days out of water once their waterbody has dried, but only if suitable shelter is available, such as wet mud or moisture under rocks and leaves (Black 1974; Downie and Smith 2003; Venturelli et al. 2021). Yet, there has been little exploration of the capacity for tadpoles to entirely remove themselves from the water column for survival. An exception includes species with semi (quasi) terrestrial tadpoles that are functionally aquatic but exploit thin films of water that run across substrates near water sources (Colaço and da Silva 2022; Delgadillo Méndez et al. 2024; Rivero 1961). In this study, we report on temporary habitat switching in tadpoles of the striped marsh frog, *Limnodynastes peronii* , which were found resting above the water's surface on floating aquatic vegetation. We explore the potential benefits of this behavioural choice to shift between habitats, either to reduce predation risk from aquatic predators and for respiration in hypoxic water.

## 2 Observations

### 2.1 Observation One

**FIGURE 1:**
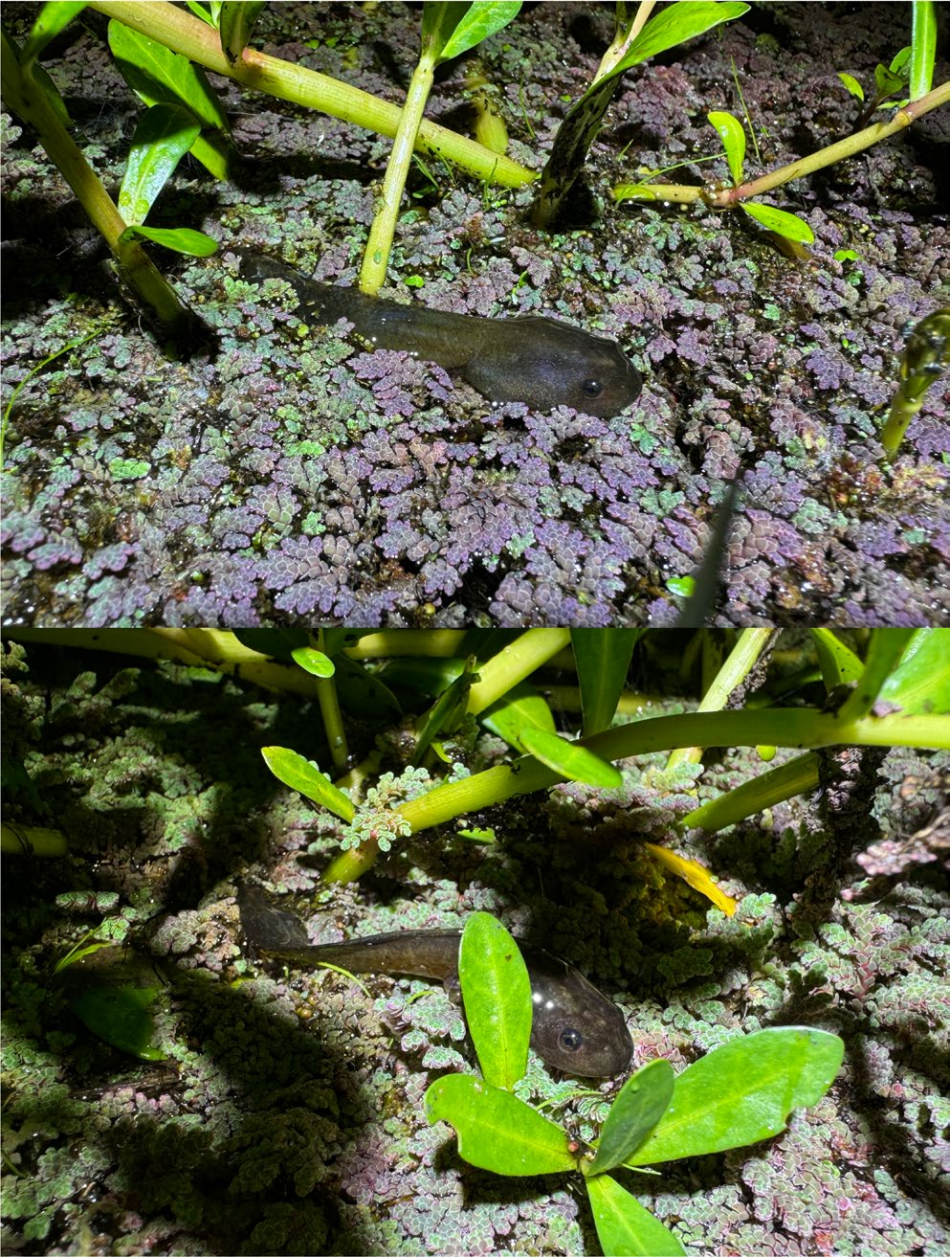
Tadpoles of the striped marsh frog, *Limnodynastes peronii*, resting above the surface of a freshwater pond on a thick mat of water fern, *Azolla* sp. Tadpoles were at a developmental stage far proceeding metamorphosis, with no indication of limb formation.

As part of nocturnal amphibian surveys on Kooragang Island in September 2024, we detected two tadpoles resting above the water's surface on floating aquatic vegetation of a freshwater pond approximately 0.35 ha in surface area (32.83427°S, 151.69884°E) (Figure 1). The primary floating plant detected at this site was water fern, *Azolla* sp., which had formed a thick mat across a majority of the surface of the pond, from the edge of the water to a small section of open water in the middle of the waterbody. The tadpoles were approximately 2–3 m from the shoreline and 5 m apart and were observed separately one after the other.

During the observational period, both tadpoles remained motionless on the *Azolla* mat for periods of up to 10–15 s, with no indication of tail motions that would suggest a struggle to re‐enter the water beneath. However, when these individuals were lightly prodded, they immediately descended back into the water column (Video 1). This occurred via rapid side‐to‐side whipping/thrashing motions of their tails that allowed them to physically push against the surface of the *Azolla* to jump and move forward, before using their heads to dig into the mat to reach the water underneath.

**VIDEO 1:** Video recording of *Limnodynastes peronii* tadpoles resting on *Azolla* mats and then rapidly descending into the water column when prodded. Video content can be viewed at https://onlinelibrary.wiley.com/doi/10.1002/ece3.71057

**FIGURE 2:**
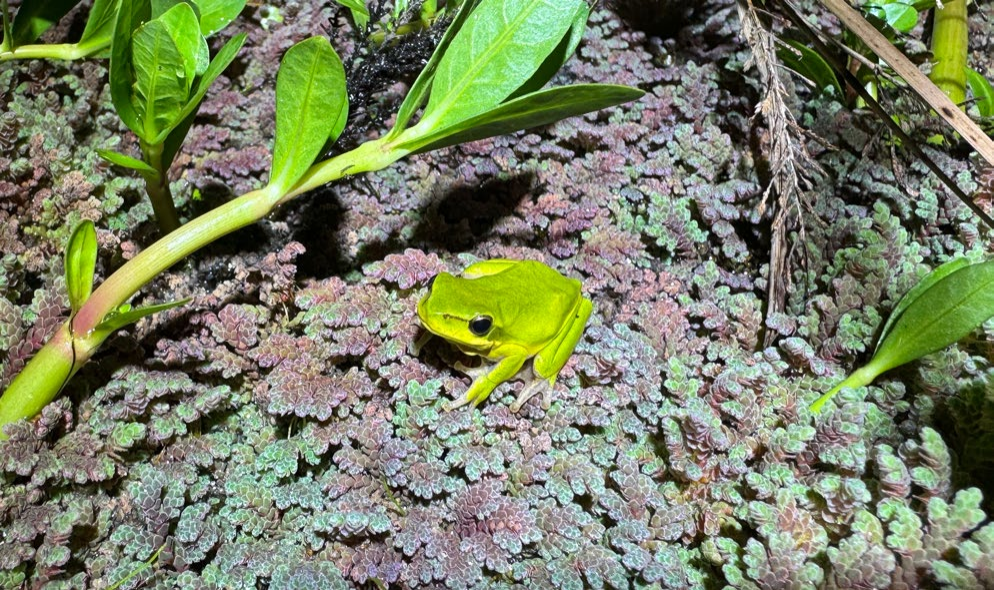
An adult *Litoria fallax* resting on a floating mat of water fern, *Azolla* sp., at a freshwater pond on Kooragang Island.

Both individuals were tadpoles of a ground frog species, likely *Lim. peronii*, based on their black colouration and morphology that is distinct from two co‐occurring tree frog species ( *Litoria peronii* and *Litoria fallax* ) that were also seen and/or heard at the pond (Anstis 2018). The only other commonly observed ground frog species on Kooragang Island are *Limnodynastes tasmaniensis* and *Crinia signifera* , yet only calling from *Lim. peronii* males was detected at the pond. Neither individual possessed front or hind limbs, and no limb nubs were visible at a distance, suggesting they were between Gosner stages 26 and 30 (Gosner 1960), with snout to tail lengths between 2 and 3 cm. More detailed recordings of tadpole size and developmental stage could not be achieved as prodding the individuals to test whether they were stuck on the *Azolla* mat led them to rapidly escape into the water column. We also detected two other animals using the *Azolla* mats, including fishing spiders from the genus *Dolomedes* and adult *L. fallax* (Figure 2). The introduced eastern mosquitofish, *Gambusia holbrooki* , was detected in the water, as well as additional tadpoles that were likely also *Lim. peronii*, although densities of both were not measured and difficult to assess visually due to the near total cover of the water's surface with floating vegetation.

### 2.2 Observation Two

**FIGURE 3:**
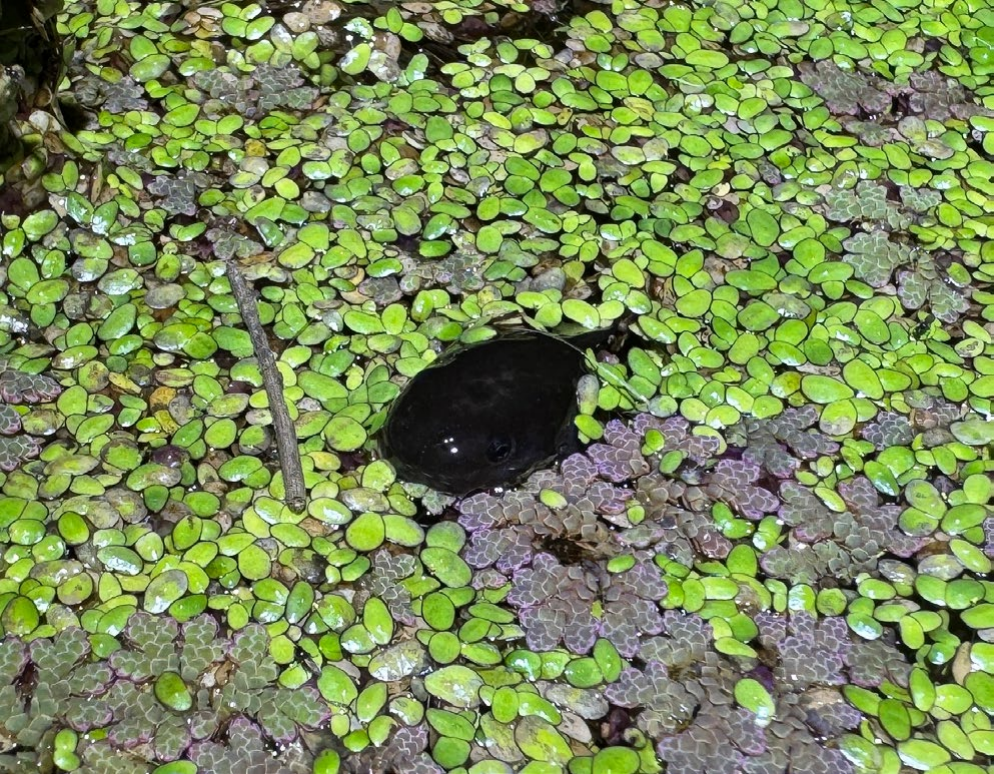
Tadpole of the striped marsh frog, *Limnodynastes peronii*, resting partially out of the water on duckweed, *Lemna minor* .

During additional surveys in November 2024, we detected another tadpole showcasing similar behaviour to those in our original observation (Figure 3). In a separate freshwater pond on Kooragang Island (32.83495°S, 151.70388°E) that was approximately 0.004 ha in surface area, we observed a *Lim. peronii* tadpole partially beached on the surface of the water on a layer of floating vegetation that was primarily duckweed but interspersed with *Azolla*. The body of the tadpole was exposed and on a slight incline, causing the tail to be almost entirely submerged in water. No limbs or limb buds were observed, suggesting the tadpole was at a Gosner stage of 26–30. The tadpole immediately descended into the water column when lightly prodded.

## 3 Discussion

Our observations indicate that *Lim. peronii* tadpoles may perform habitat switching by moving out of the water and use floating vegetation as a resting surface when it is of sufficient thickness to support their weight. This is different to the emergence that typically occurs in some amphibians at metamorphosis, where a loss of gills as individuals transition from tadpoles to juveniles is accompanied by the movement onto emergent vegetation or the shoreline to prevent drowning (Crespi and Warne 2013; Stehouwer 1988). Indeed, the tadpoles we observed were at a stage of development far too premature for metamorphosis to have occurred, given the near‐total lack of limb formation and lack of tail reabsorption (Gosner 1960). Additionally, there was no indication that the tadpoles had become stuck on the floating vegetation mats, given the lack of writhing behaviour and their ability to rapidly return to the water when disturbed. Instead, it appears that the aquatic tadpoles of some amphibian species may have the potential to temporarily exploit systems outside of the water column for resource gain and may not be entirely confined to water prior to metamorphosis.

There has been little previous investigation into the ability or desire of aquatic tadpoles to entirely remove themselves from the water column on purpose. An exception is semi‐terrestrial tadpoles that can survive and move along films of water. For example, *Leptodactylus lithonaetes* tadpoles have been recorded moving across wet moss on rocks (Barrientos et al. 2018), between pools using thin layers of water (Rivero 1961), and remaining in shallow water with parts of their dorsum exposed (Delgadillo Méndez et al. 2024). This is in contrast to circumstances that arise in waterbodies that have started to dry where tadpoles unintentionally become stuck out of water (Gould et al. 2022). Other behaviors expressed by tadpoles indicate that there is some ability to move at least partially out of the water and exploit resources above the surface. For example, most tadpoles participate in air‐breathing by piercing the surface of the water with the front of their faces (breach breathing) or drawing in air bubbles (bubble‐sucking), to obtain oxygen and/or for lung development (Pronych and Wassersug 1994; Schwenk and Phillips 2020; Wells 2007). However, these behaviors are not true expressions of habitat switching, in contrast to moving out of the water column.

Moving onto floating vegetation could be a form of adaptive beaching behaviour that is undertaken for some sort of benefit as opposed to random. We hypothesise that it could be a strategy of habitat switching to avoid aquatic threats, namely predators that cannot move beyond the surface of the water themselves. We detected the presence of *Gambusia* in the pond where both tadpoles were found, which are known to be a voracious predator of amphibian offspring including eggs and tadpoles (Komak and Crossland 2000). Temporarily moving out of the water would protect tadpoles from immediate, aquatic threats such as co‐occurring predatory fish, akin to aquatic mammals that move onto ice sheets and land to avoid attacks by killer whales (Vila et al. 2008). However, as tadpoles are morphologically adapted to locomotion in water (Wassersug 1989), they may be at an increased risk of predation by terrestrial predators while beached, such as spiders (Jara 2008), which we also detected moving across the *Azolla* mats. Our observations could suggest that there are opportunities for tadpoles to move out of water to distance themselves from aquatic predators, expanding the choices that can be made in terms of spatial refuges and microhabitat site selection when responding to such threats. This may be restricted to tadpoles of a certain size and thus stage, as the smaller surface area to volume ratio of larger individuals may reduce their rate of evaporative water loss when large portions of their bodies are out of water (e.g., Newman and Dunham 1994), although this requires further investigation.

Beaching could also be a strategy for tadpoles to acquire oxygen from the air when the water has become hypoxic (Burggren and Infantino 1994). This is possible as amphibian tadpoles can respire using a variety of strategies dependent on developmental stage, including in the water via gills and cutaneous respiration, as well as lungs that start to develop and become functional before metamorphosis occurs (Phillips et al. 2020; Wells 2007). Floating vegetation across the surface of a waterbody may (i) prevent sunlight from reaching submerged plants and algae (Rabaey and Cotner 2022), thereby reducing their oxygen output into the surrounding water column that would limit gas exchange via the skin and gills, and (ii) prevent tadpoles from air‐breathing via the lungs if the water's surface becomes covered, which could affect lung development and metamorphosis (Pronych and Wassersug 1994). Our observations occurred at ponds which were almost entirely covered by *Azolla* and/or duckweed, which may have triggered the need for breaching by reducing water oxygenation, although water quality was not directly measured. If this was indeed the case, we would have expected to see more tadpoles out of the water, but this may have been due to insufficient survey intensity or a low density of tadpoles within the pool, or it may suggest that beaching was only periodically performed by individuals. Nevertheless, a unique situation arises where the formation of vegetation mats may not only trigger the need for beaching behaviour by creating hypoxic conditions, but it also provides the necessary structures to support resting above the water for air breathing. The hypotheses we mention could be tested in the laboratory by placing tadpoles at different stages under varying levels of hypoxia and in the presence of different densities of floating materials, to assess critical thresholds where breaching behaviour is triggered and the materials that may provide the necessary support for it to be performed.

In general, the surface of the water represents a barrier to movement for aquatic tadpoles. Yet, our observations provide evidence that such tadpoles may have the capacity to temporarily escape the water column, as an adaptive behaviour that potentially confirms some sort of benefit. However, additional observations are required to determine whether this is a random behaviour exhibited by a few individuals or a trait expressed more generally in the species, to confirm whether it does indeed improve survival, and the length of time tadpoles can remain breached. This compliments previous observations on semi‐terrestrial tadpoles and terrestrial nesting species with tadpoles that have the capacity to survive out of water for extended periods, showing that movement out of water is possible for at least some tadpoles that are water obligates and without an evolutionary history of terrestriality.

## Author Contributions

**John Gould:** conceptualization (lead), investigation (lead), visualization (lead), writing – original draft (lead), writing – review and editing (equal). **Claire Larkin:** investigation (supporting), writing – original draft (supporting), writing – review and editing (equal). **Colin McHenry:** supervision (lead), writing – original draft (supporting), writing – review and editing (equal).

## Conflicts of Interest

The authors declare no conflicts of interest.

## Data Availability

The authors have nothing to report.
